# Induction of zinc finger protein RNF6 auto-ubiquitination for the treatment of myeloma and chronic myeloid leukemia

**DOI:** 10.1016/j.jbc.2022.102314

**Published:** 2022-08-01

**Authors:** Haixia Zhuang, Ying Ren, Chenyu Mao, Yueya Zhong, Zubin Zhang, Biyin Cao, Yuming Zhang, Jinqi Huang, Guoqiang Xu, Zhenqian Huang, Yujia Xu, Xinliang Mao

**Affiliations:** 1 Department of Hematology, The First Affiliated Hospital & Guangdong Key Laboratory of Protein Modification and Degradation, School of Basic Medical Sciences, Guangzhou Medical University, Guangzhou, Guangdong, China; 2 Department of Pharmacology, College of Pharmaceutical Sciences, Soochow University, Suzhou, Jiangsu, China; 3 School of Medicine, Southern University of Science and Technology, Shenzhen, Guangdong, China; 4 Department of Hematology, Hematology Research Institute, Affiliated Hospital of Guangdong Medical University, Zhanjiang, Guangdong, China

**Keywords:** USP7, RNF6, auto-ubiquitination, leukemia, myeloma, CHX, cycloheximide, IB, immunoblot, IP, immunoprecipitation, MM, multiple myeloma, PBX1, Pre-B-Cell Leukemia Transcription Factor 1, RING, Really Interesting New Gene, SDS-PAGE, sodium dodecyl sulfate–polyacrylamide gel electrophoresis, UBE1, Ubiquitin E1 enzyme

## Abstract

The zinc finger ubiquitin ligase RNF6 has been proposed as a potential therapeutic target in several cancers, but understanding its molecular mechanism of degradation has been elusive. In the present study, we find that RNF6 is degraded *via* auto-ubiquitination in a manner dependent on its Really Interesting New Gene (RING) domain. We determine that when the RING domain is deleted (ΔRING) or the core cysteine residues in the zinc finger are mutated (C632S/C635S), the WT protein, but not the ΔRING or mutant RNF6 protein, undergoes polyubiquitination. We also identify USP7 as a deubiquitinase of RNF6 by tandem mass spectrometry. We show that USP7 interacts with RNF6 and abolishes its K48-linked polyubiquitination, thereby preventing its degradation. In contrast, we found a USP7-specific inhibitor promotes RNF6 polyubiquitination, degradation, and cell death. Furthermore, we demonstrate the anti-leukemic drug Nilotinib and anti-myeloma drug Panobinostat (LBH589) induce RNF6 K48-linked polyubiquitination and degradation in both multiple myeloma (MM) and leukemia cells. In agreement with our hypothesis on the mode of RNF6 degradation, we show these drugs promote RNF6 auto-ubiquitination in an *in vitro* ubiquitination system without other E3 ligases. Consistently, reexpression of RNF6 ablates drug-induced MM and leukemia cell apoptosis. Therefore, our results reveal that RNF6 is a RING E3 ligase that undergoes auto-ubiquitination, which could be abolished by USP7 and induced by anti-cancer drugs. We propose that chemical induction of RNF6 auto-ubiquitination and degradation could be a novel strategy for the treatment of hematological malignancies including MM and leukemia.

The zinc finger protein RNF6 is an oncogenic ubiquitin ligase that has been overexpressed in various cancers including multiple myeloma (MM) cells (1) and leukemia cells (2). In MM cells, RNF6 as a ubiquitin ligase binds to and mediates K63-linked polyubiquitination toward the glucocorticoid receptor and promotes its oncogenic transcriptional activity, therefore contributing to MM cell survival and drug resistance (1). In leukemia cells, RNF6 is upregulated by the Pre-B-Cell Leukemia Transcription Factor 1 (PBX1) and promotes leukemia progression (2). Knockdown of RNF6 leads to shrinkage of human leukemia xenografts in mice. Moreover, downregulation of RNF6 by the natural product saponins from *Paris forrestii* induces leukemia cell apoptosis (3). All these studies suggest RNF6 could be a potential therapeutic target for both MM and leukemia.

RNF6 belongs to the C2H2-type zinc finger ubiquitin ligases containing a Really Interesting New Gene (RING) motif and this class of ubiquitin ligases can mediate themselves ubiquitination or auto-ubiquitination (4). A number of such ubiquitin ligases have been well elucidated including XIAP (5), TRAF6 (6), TRIM26 (7), and RNF115 (7). Similar to general ubiquitination, auto-ubiquitination can be classified as mono-ubiquitination or various polyubiquitination types, therefore leading to self-degradation or functional modification. It is reported that mono-ubiquitination of TRIM26 by itself occurs upon viral infection, thus leading to the activation of TBK1 innate antiviral immune response (7). In contrast, XIAP undergoes degradation upon self-ubiquitination induced by its antagonist, therefore leading to the activation of Caspase-3 and cancer cell apoptosis (5). Given that auto-ubiquitination is a characteristic of RING E3 ligases, whether RNF6 could undergo auto-ubiquitination and how this auto-ubiquitination is modulated remains elusive. It will be of interest to know whether induction of RNF6 auto-ubiquitination benefits the treatment of hematological malignancies.

In the present study, we found that RNF6 is degraded *via* the proteasomes upon self-directed polyubiquitination. Moreover, we identified that the ubiquitin-specific protease USP7 stabilizes RNF6 by preventing its auto-ubiquitination. Inhibiting USP7 leads to RNF6 degradation and cell apoptosis. Furthermore, we found that anti-cancer drugs can trigger RNF6 with K48-linked auto-ubiquitination and proteasomal degradation. This study provides an in-depth understanding on the modulation of RNF6 auto-ubiquitination and initiated the concept that chemical induction of auto-ubiquitination represents a novel strategy for the treatment of leukemia and myeloma.

## Results

### RNF6 undergoes auto-ubiquitination and proteasomal degradation

**Figure 1:**
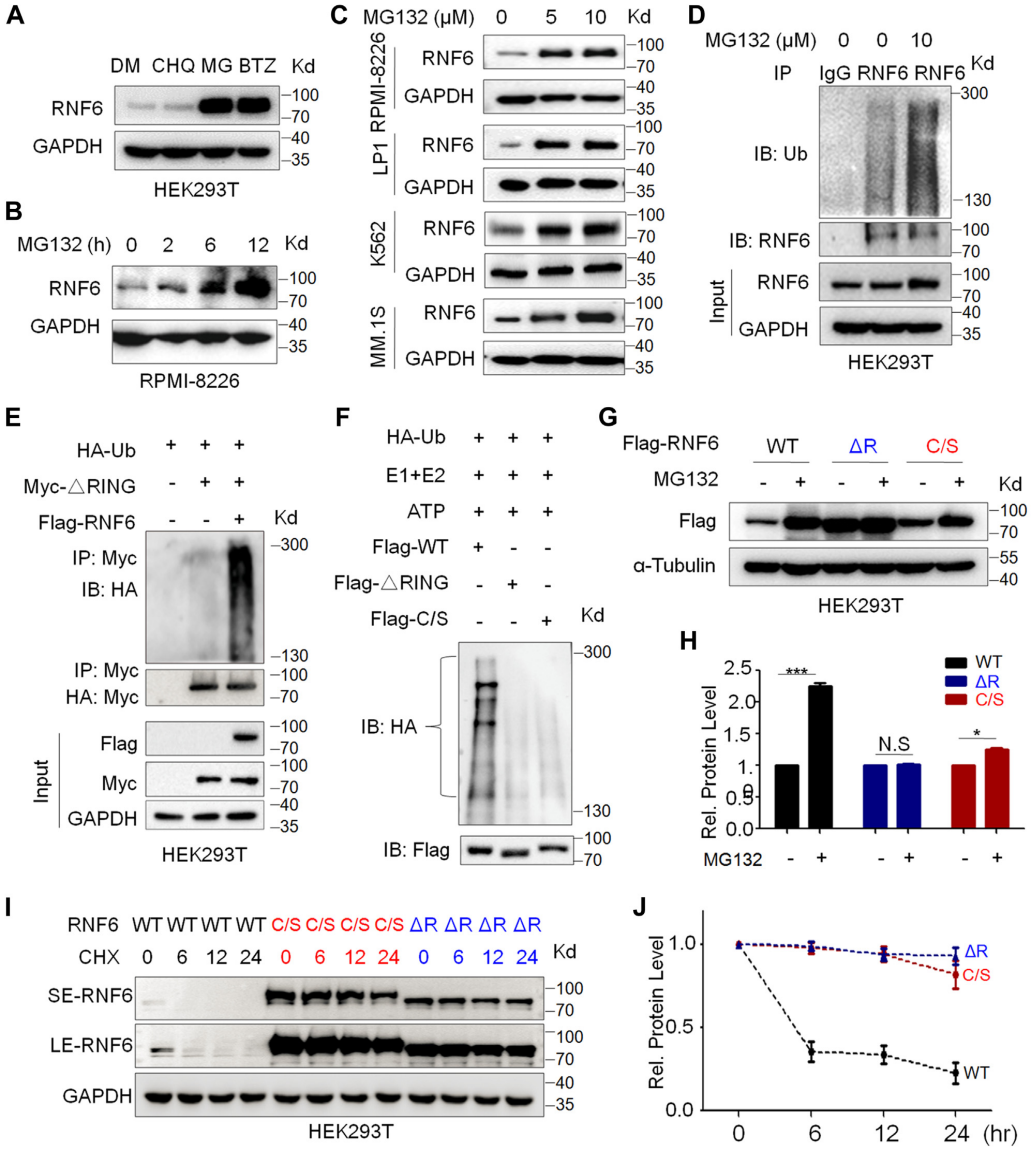
**RNF6 turnover is processed *via* the ubiquitin-proteasomal pathway dependent on its auto-ubiquitination.***A*, HEK293T cells were treated with proteasomal inhibitors (BTZ, MG132) or lysosomal inhibitor (chloroquine, CHQ) for 12 h followed by IB assays. *B*, RPMI-8226 cells were treated with MG132 at 10 μM for the indicated periods before being subjected to IB assays as indicated. *C*, RPMI-8226, LP1, MM.1S, and K562 cells were treated with MG132 at the increasing concentrations for 12 h, followed by IB assay. *D*, HEK293T cells were treated with MG132 for 12 h. Cell lysates were then subjected to immunoprecipitation (IP) and IB assay. *E*, HEK293T cells were cotransfected with HA-Ub and Myc-ΔRING or Flag-RNF6 plasmids. After 48 h, cells were harvested and subjected to IP/IB assays. *F*, purified WT, ΔRING, or C/S RNF6 proteins were incubated with reaction buffer containing HA-Ub, E1, E2, and ATP for 30 min, followed by IB assays as indicated. *G*, HEK293T cells were transfected with WT, ΔRING, or C to S mutant (C/S) RNF6 for 24 h, followed by MG132 treatment for 12 h before being subjected to IP/IB assays. *H*, the RNF6 expression level from (*G*) was evaluated by a densitometric assay. *I*, HEK293T cells were transfected with WT, C/S, or ΔRING RNF6 for 24 h, followed by CHX treatment at indicated periods before being harvested and subjected to IB assays as indicated. *J*, the density of RNF6 blots from (*I*) was evaluated by densitometric assay. N.S., not significant. ∗*p* < 0.05; ∗∗∗*p* < 0.001. IB, immunoblot; LE, long exposure; MM, multiple myeloma; RING, Really Interesting New Gene; SE, short exposure.

Given that RNF6 is a member of the RING family E3 ligases featured with auto-ubiquitination and degradation (4), we first examined whether RNF6 could be degraded *via* the proteasomes. To this end, we first evaluated RNF6 stability in HEK293T cells that were treated with proteasomal inhibitors or lysosomal inhibitors for 12 h, and the subsequent assays demonstrated that RNF6 protein was markedly increased by proteasome inhibitors (MG132 and bortezomib) but not by a typical lysosomal inhibitor chloroquine (Fig. 1*A*). Moreover, the RNF6 protein stability was increased by proteasomal inhibition in a concentration- and time-dependent manner (Fig. 1, *B* and *C*). Furthermore, MG132 also strikingly accumulated RNF6 polyubiquitination in a concentration-dependent manner (Fig. 1*D*), suggesting RNF6 was modified by polyubiquitination and it was processed in proteasomes.

Given that RNF6 is a RING domain E3 ligase, we wondered whether RNF6 polyubiquitination is self-directed. To this end, we generated a ΔRNF6 construct lacking the RING domain that determines E3 ligase activity and evaluated its ubiquitination level. We found that in the presence of intact RNF6, ΔRNF6 could be polyubiquitinated (Fig. 1*E*), but no polyubiquitination was observed on ΔRNF6 in the absence of intact RNF6, suggesting RNF6 might direct the polyubiquitination of ΔRNF6; in other words, RNF6 might undergo auto-ubiquitination. To confirm this hypothesis, we further constructed a RNF6 double mutant with C632S/C635S (RNF6^C/S^) to disrupt the zinc finger scaffold, thereby inactivating RNF6 E3 ligase activity. We purified wtRNF6, ΔRNF6, or RNF6^C/S^ and further measured their ubiquitination levels in a cell-free ubiquitination system in the absence of any other E3 ligases. The result showed that wtRNF6 but not ΔRNF6 or RNF6^C/S^ was polyubiquitinated (Fig. 1*F*), further confirming the conclusion that RNF6 undergoes auto-ubiquitination. Subsequently, we measured the protein stability of RNF6 and its mutants in the presence of MG132 for 24 h and found that MG132 stabilized wtRNF6 but not its RING-deleting counterparts (Fig. 1, *G* and *H*). Moreover, when cycloheximide (CHX), an inhibitor of protein synthesis *de novo*, was added, wtRNF6 was almost completely degraded within 6 h, but ΔRNF6 remained unchanged within 24 h (Fig. 1, *I* and *J*), further suggesting RNF6 stability relied on its RING domain–related E3 ligase activity. However, the C/S mutant was found to have a slight increase by MG132 in 24 h (Fig. 1, *G* and *H*), which was confirmed in the CHX chase assay (Fig. 1, *I* and *J*). To be noted, C/S mutant was not markedly altered in 12 h (Fig. 1, *I* and *J*), suggesting that the C/S mutant might still undergo degradation in proteasomes with a yet-to-know mechanism. All the above results therefore collectively demonstrated that RNF6 undergoes self-directed polyubiquitination and degradation in proteasomes.

### USP7 binds to RNF6 in leukemia and myeloma cells

**Figure 2:**
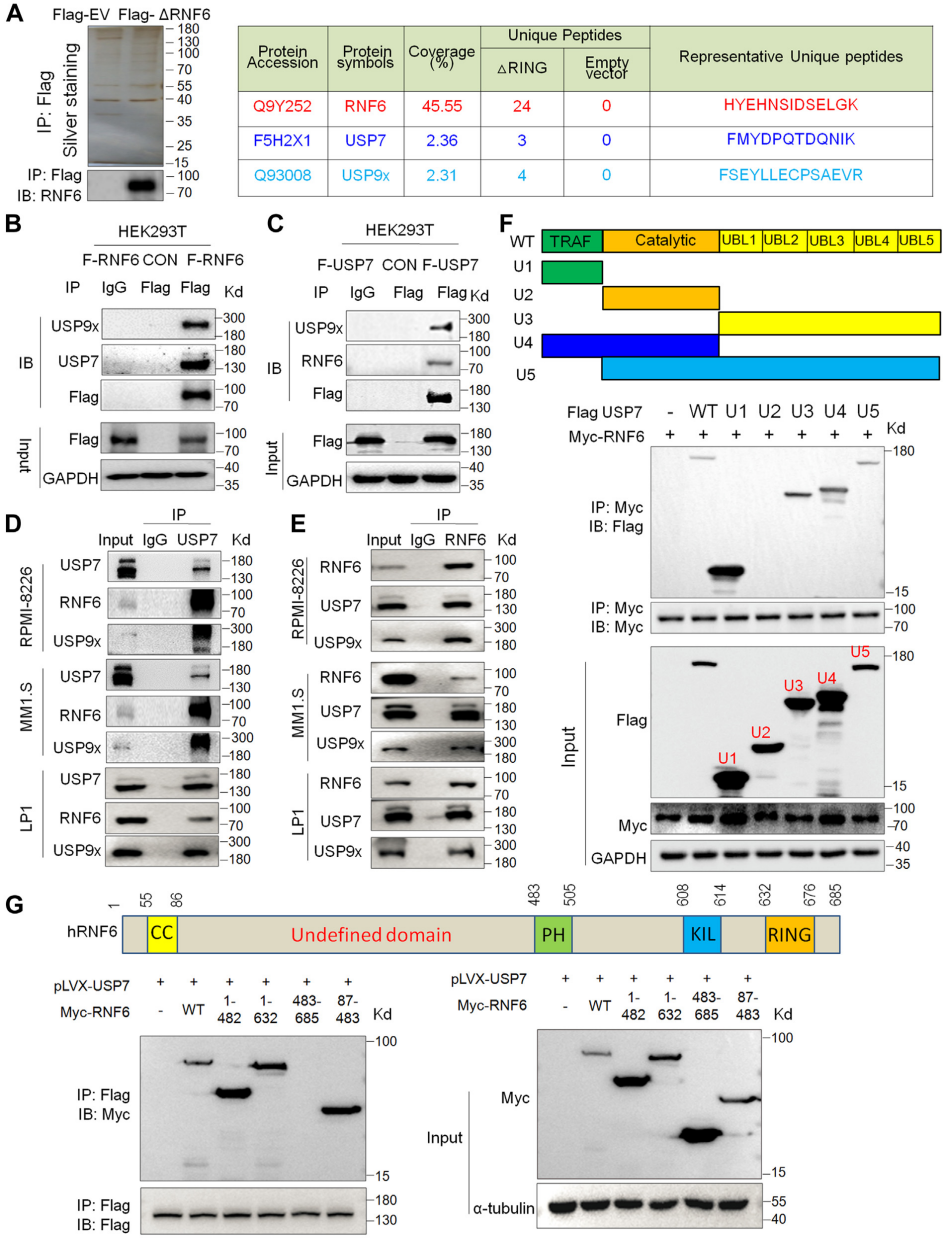
**Both USP7 and USP9x interact with RNF6.***A*, the RNF6△RING plasmid or the empty vector was transfected into HEK293T cells, followed by immunoprecipitation using an anti-Flag antibody. The protein precipitates were then subjected to SDS-PAGE and silver staining (*Left*). The IP results were subjected to an IB assay with an anti-Flag antibody (*Left*). The gel bands were sliced for in-gel digestion with trypsin. The peptides were then recovered and purified for HPLC-MS/MS. The results showed that deubiquitinases USP7 and USP9x were identified by MS (*Right*). *B*, HEK293T cells were transfected with a Flag-RNF6 (F-RNF6) plasmid, followed by IP with an anti-IgG or anti-Flag antibody and subsequent IB assays. *C*, HEK293T cells were transfected with a Flag-USP7 (F-USP7) plasmid, followed by IP with IgG or anti-Flag antibody and subsequent IB assays. *D*, cell lysates from various cell lines were incubated with IgG or anti-USP7 antibody overnight, followed by IB with specific antibodies as indicated. *E*, cell lysates from various cell lines were incubated with IgG or anti-RNF6 antibody overnight, followed by IB with specific antibodies as indicated. *F*, the USP7 truncates were constructed as indicated, followed by cotransfection with Myc-RNF6 plasmids. Thirty-six hours later, cell lysates were collected for IP/IB as indicated. *G*, the RNF6 truncates were constructed as indicated (*Upper panel*), followed by cotransfection with Flag-USP7 plasmids. Thirty-six hours later, cell lysates were collected for IP/IB as indicated. *Right*, the IP/IB result, *Left*, the input result. CON, control; EV, empty vector; IB, immunoblot; IP, immunoprecipitation; MS, mass spectroscopy, RING, Really Interesting New Gene; SDS-PAGE, sodium dodecyl sulfate–polyacrylamide gel electrophoresis.

The above study clearly demonstrated that RNF6 undergoes auto-ubiquitination. Given that protein ubiquitination is a dynamic and reversible process, we wondered whether there is a deubiquitinase that modulates RNF6 auto-ubiquitination. To this end, we performed a HPLC-coupled tandem mass spectrometry assay against ΔRNF6-interactomes (given ΔRNF6 is more stable), from which two deubiquitinases USP7 and USP9x were identified with more than two unique peptides in the RNF6-interacting proteome from the ΔRNG but not in the empty-vector–transfected cells (Fig. 2*A*). To confirm the interaction between RNF6 and the Dubs, we overexpressed Flag-RNF6 or Flag-USP7 in HEK293T cells, respectively, followed by immunoprecipitation (IP) with a specific antibody against Flag, the subsequent immunoblot (IB) showed that both USP7 and USP9x proteins were found in the RNF6 immunoprecipitates (Fig. 2*B*) and both RNF6 and USP9x were present in the USP7 immunoprecipitates (Fig. 2*C*). Moreover, this interaction was recapitulated in multiple MM cell lines (Fig. 2, *D* and *E*). Lastly, we examined the detailed interaction between USP7 and RNF6 and found that the N-terminal TRAF or the C-terminal UBL domains were critical for USP7 to interact with RNF6 (Fig. 2*F*). We also found that RNF6 interacted with USP7 *via* its undefined domain (aa. 87–482) (Fig. 2*G*).

### USP7 deubiquitinates the RNF6 protein

**Figure 3:**
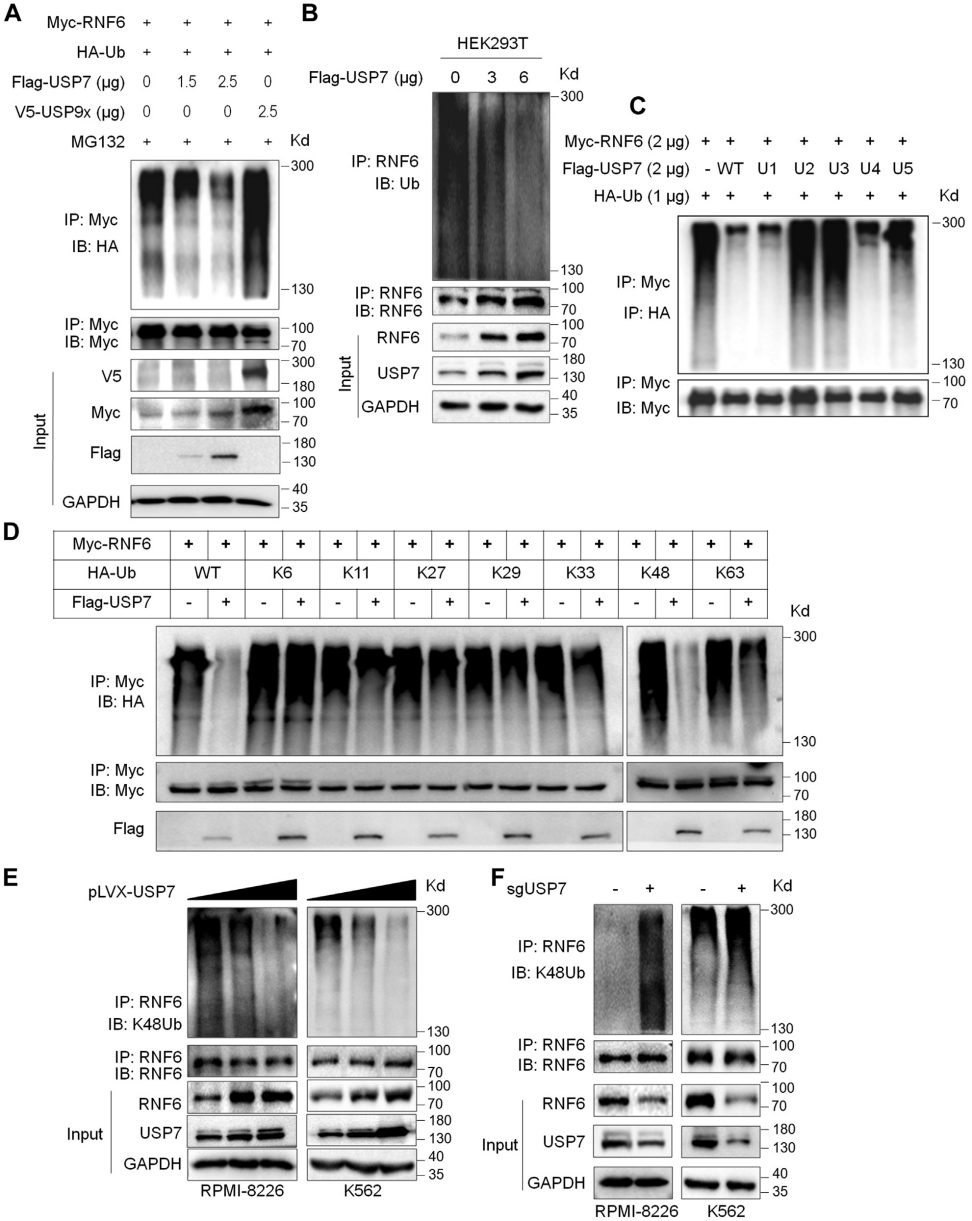
**USP7 prevents RNF6 from K48-linked polyubiquitination.***A*, RNF6 (2 μg) and Ub (1.5 μg) plasmids were cotransfected into HEK293T cells with a USP7 or USP9x plasmid for 24 h, followed by 10 μM MG132 treatment for 12 h. The cell lysates were then prepared for IP and IB assays as indicated. *B*, Flag-USP7 plasmids were transfected into HEK293 cells for 48 h, followed by MG132 treatment and subjected to IP/IB assays as indicated. *C*, USP7 truncates as shown in Figure 2*F* were cotransfected into HEK293T cells with RNF6 and Ub plasmids for 24 h, followed by MG132 treatment and IP/IB assays. *D*, RNF6 was cotransfected into HEK293T cells with mutated Ub as indicated in the presence or absence of USP7 for 24 h, and cells were then treated with MG132 for 10 h before being subjected to IP/IB assays as indicated. *E*, lentiviral USP7 was subjected to infect RPMI-8226 and K562 cells for 96 h, followed by IP/IB assays as indicated. *F*, USP7 was knocked out by CRISP-Cas9 technology for 96 h, followed by IP/IB assays as indicated. IB, immunoblot; IP, immunoprecipitation.

Given that both USP7 and USP9x interact with RNF6, we wondered whether these two Dubs deubiquitinated RNF6. To this end, RNF6 was cotransfected with USP7 or USP9x into HEK293T cells, followed by IP/IB assays. The results revealed that USP7 but not USP9x markedly reduced the polyubiquitination level of RNF6 (Fig. 3*A*). In contrast, USP9x increased the polyubiquitination levels of RNF6 (Fig. 3*A*). This finding suggested that USP7 but not USP9x was a potential Dub of RNF6. We thus subsequently evaluated the effects of USP7 on RNF6 ubiquitination. As shown in Figure 3*B*, USP7 downregulated the ubiquitination level of RNF6 in a concentration-dependent manner. A previous study has shown that a specific domain in USP7 might be sufficient to stabilize its substrate protein (8). To find out which domain was critical for USP7 to deubiquitinate RNF6, we structured a series of USP7 truncates (Fig. 2*F*) and these truncates were subjected to co-transfection into HEK293T cells with RNF6. The resultant IP/IB assay demonstrated that the catalytic domain was not sufficient to deubiquitinate RNF6 (Fig. 3*C*); it must act together with either the TRAF or the UBL domain (Fig. 3*C*). Interestingly, the truncate with the TRAF domain remained equal deubiquitinase activity comparable to the WT form (Fig. 3*C*).

It is known that there are several types of ubiquitination upon the lysine residue in ubiquitin; to find out which type of ubiquitination could be prevented by USP7, we cotransfected USP7, RNF6, and individual Ub plasmids with single lysine residues into HEK293T cells, and the subsequent IP/IB assay revealed that RNF6 was processed with high polyubiquitination in the presence of Ub. Notably, USP7 abolished RNF6 from K48- but not other Ub-linked polyubiquitination (Fig. 3*D*), indicating that RNF6 might mainly undergo K48-linked auto-ubiquitination and USP7 deubiquitinates RNF6 for its K48-linked ubiquitination. To confirm this hypothesis, we next examined the endogenous K48-linked polyubiquitination of RNF6 in both the MM cell line RPMI-8226 and chronic myelogenous leukemia CML cell line K562. The specific IP/IB assays indicated that USP7 downregulated RNF6 for its K48-linked polyubiquitination in both cell lines in a concentration-dependent manner (Fig. 3*E*). In accordance with this finding, when USP7 was knocked out by its specific sgRNA, RNF6 was found to be modified with increased K48-linked polyubiquitination (Fig. 3*F*). Therefore, these results collectively demonstrated that RNF6 might undergo K48-linked auto-ubiquitination that could be abolished by the Dub USP7.

### USP7 stabilizes RNF6 at the protein level

**Figure 4:**
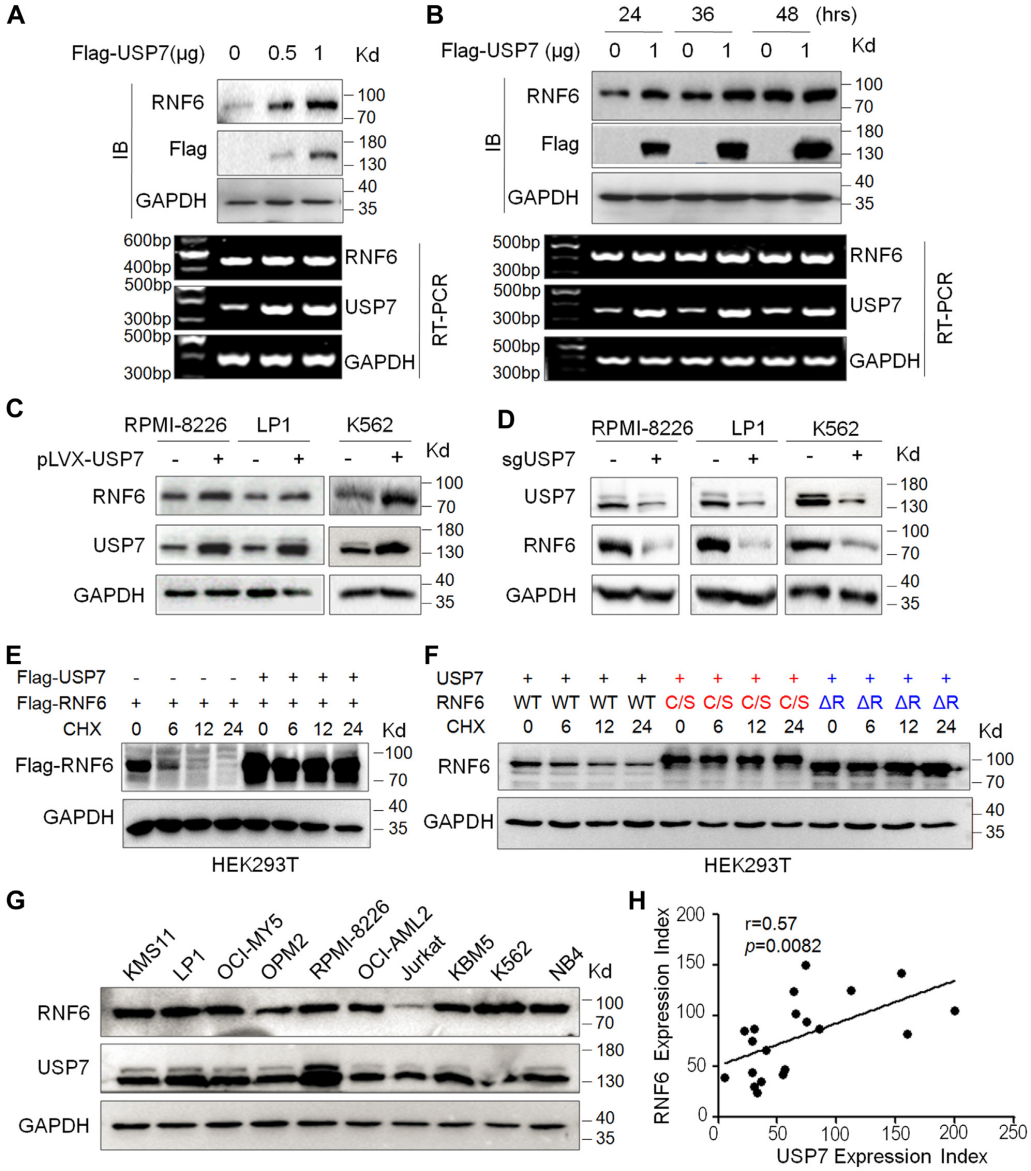
**USP7 stabilizes RNF6 protein but displays no activity on its mRNA.***A*, HEK293T cells were transfected with the increasing concentrations Flag-USP7 plasmids for 48 h, followed by IB and RT-PCR assays. *B*, HEK293T cells were transfected with Flag-USP7 plasmids for the indicated periods, followed by IB and RT-PCR assays. *C*, various MM and CML cell lines were infected with lentiviral USP7 for 96 h, followed by cell lysate preparation and IB assays as indicated. *D*, USP7 was knocked out of indicated cells for 96 h, followed by IB assays as indicated. *E*, HEK293T cells were transfected with WT RNF6 (Flag-RNF6) with or without USP7 for 24 h, followed by CHX treatment. Cells were collected for the IB assay at the indicated periods after CHX treatment. *F*, HEK293T cells were transfected with USP7 and WT or mutant RNF6 plasmids for 24 h, followed by CHX treatment for the indicated periods before cell lysates were prepared for IB assays. *G*, cell lysates from various cell lines were subjected to IB against RNF6 and USP7. *H*, bone marrow cells from MM patients (n = 20) were collected for IB assays against RNF6 and USP7. The correlation analysis of RNF6 and USP7 expression in MM patients’ bone marrow was presented after normalization to α-tubulin expression. The Pearson correlation coefficient was 0.57 with *p* < 0.01. CHX, cycloheximide; IB, immunoblot; MM, multiple myeloma.

The above studies have demonstrated that USP7 but not USP9x decreases RNF6 for its K48-linked polyubiquitination that is necessary for protein degradation in proteasomes; therefore, we wondered whether USP7 prevents RNF6 from degradation *via* the ubiquitin-proteasomal pathway. To this end, Flag-USP7 was transfected into cells followed by evaluating RNF6 at the protein and mRNA levels. The results showed that USP7 failed to alter RNF6 mRNA but upregulated its protein in a concentration- and time-dependent manner (Fig. 4, *A* and *B*). These findings were consistent with the previously mentioned study that USP7 prevented RNF6 from K48-linked polyubiquitination in association with protein stability (Fig. 3). The effects of USP7 on RNF6 protein were further recapitulated in MM and leukemia cell lines (Fig. 4*C*). When USP7 was introduced into RPMI-8226, LP1, and K562 cells, RNF6 protein was increased (Fig. 4*C*); in contrast, when USP7 was knocked out by its specific sgRNA, RNF6 protein was markedly reduced (Fig. 4*D*). To further find out whether USP7 stabilizes RNF6, we examined the effect of USP7 on the half-life of RNF6 in the presence of CHX. As shown in Figure 4*E*, RNF6 was almost completely degraded within 6 h, in a manner as shown in Figure 1*I*; however, the introduction of USP7 markedly extended its degradation time. Given that RNF6 undergoes auto-ubiquitination, we next examined the effects of USP7 on the metabolism of wtRNF6 as well as its ΔRING and C/S mutants. The results showed that USP7 stabilized the wtRNF6 protein but showed no activity to increase the protein stability of the ΔRING and C/S mutants (Fig. 4*F*), further suggesting that USP7 antagonizes RNF6 auto-ubiquitination and subsequent degradation. To further characterize the correlation between USP7 and RNF6 protein levels in MM and leukemia cells, a panel of MM and leukemia cell lines as well as primary MM bone marrow cells were subjected to IB to measure USP7 and RNF6 proteins. The results showed that the RNF6 protein level was highly correlated to USP7 levels in both types of cell lines (Fig. 4*G*) and primary cells (Fig. 4*H*). Therefore, there results demonstrated that USP7 stabilizes RNF6 protein.

### Inhibition of USP7 promotes RNF6 degradation *via* accumulating its K48-polyubiquitination

**Figure 5:**
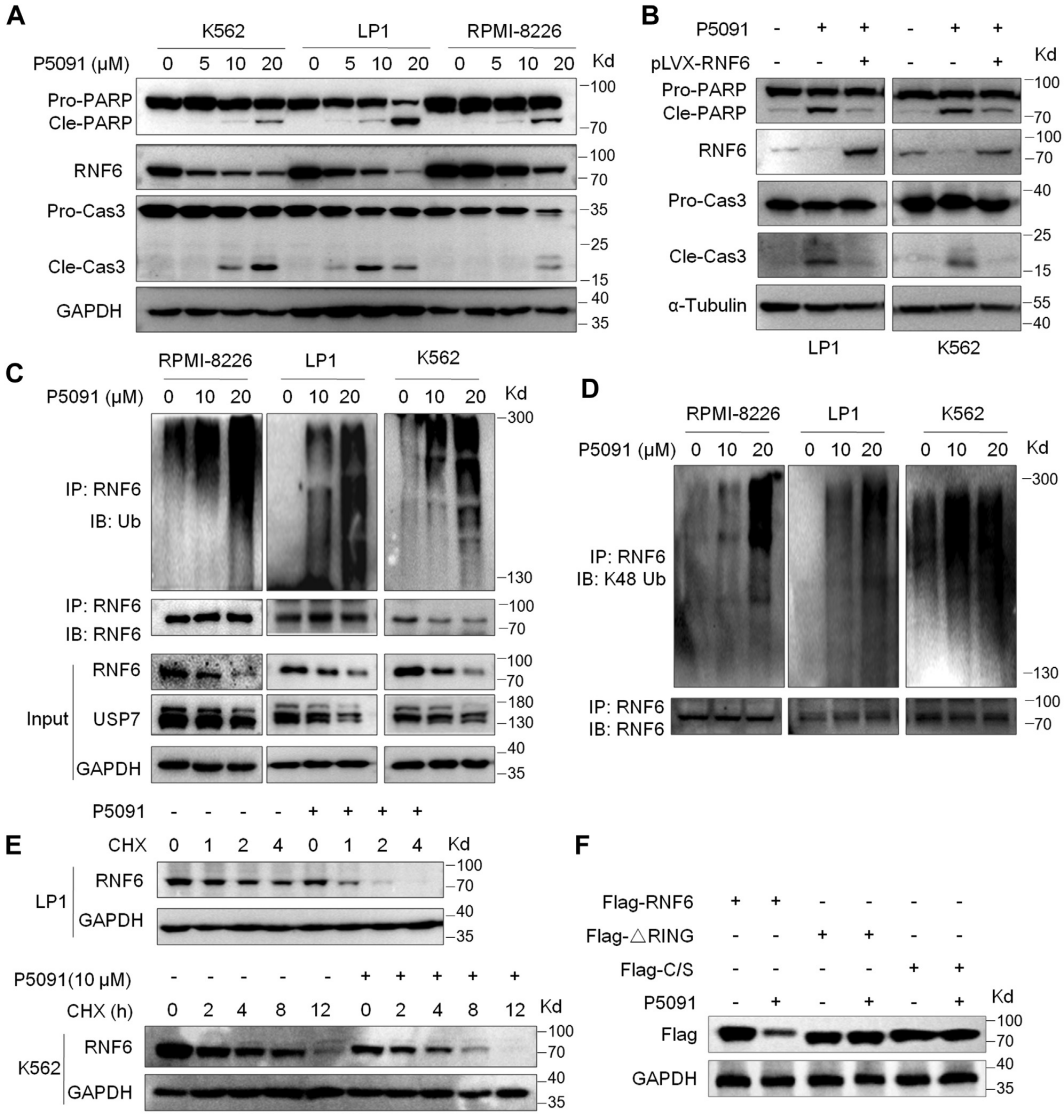
**Inhibition of USP7 leads to the accumulation of RNF6 polyubiquitination and degradation.***A*, K562, LP1, and RPMI-8226 were treated with P5091 at increasing concentrations for 24 h, followed by an IB assay for the indicated proteins. *B*, LP1 and K562 cells were infected with lentiviral RNF6 for 72 h, followed by P5091 treatment for 12 h, and cell lysates were then prepared for IB assays. *C* and *D*, RPMI-8226, LP1, and K562 cells were treated with P5091 for 12 h, followed by IP and IB assays as indicated. *E*, LP1 and K562 cells were pretreated with or without P5091(10 μM) for 12 h, followed by CHX (100 μg/ml) treatment for the indicated periods and IB assays. *F*, Flag-RNF6, ΔRING, or the C/S mutant was transfected into HEK293T cells; 24 h later, cells were treated with P5091 for 12 h and cell lysates were then subjected to IB assays against specific proteins as indicated. IB, immunoblot; IP, immunoprecipitation; RING, Really Interesting New Gene.

The previous studies have shown that RNF6 stability could be modulated by its auto-ubiquitination and USP7 acts as a Dub of RNF6 self-ubiquitination. These findings suggest that inhibition of USP7 might induce RNF6 degradation *via* the ubiquitin-proteasomal pathway. To validate this hypothesis, MM and leukemia cell lines, including RPMI-8226, LP1, and K562, were treated with P5091, a small chemical molecular inhibitor of USP7 (9). The subsequent analysis revealed that P5091 downregulated RNF6 at its protein level, along with the cleavage of PARP and Caspase-3, hallmarks of cell apoptosis (Fig. 5*A*), indicating P5091 induces MM and CML cell apoptosis that might be at least partly contributed by RNF6 degradation. To further confirm this finding, LP1 and K562 were infected with lentiviral RNF6, followed by P5091 treatment and IB assays. The results showed that RNF6 overexpression significantly abolished the action of P5091 in terms of PARP cleavage and Caspase-3 activation in both cell lines (Fig. 5*B*). Therefore, these results suggested that RNF6 degradation is highly associated with P5091 in MM and CML cell apoptosis. Given that P5091 is an inhibitor of USP7 while USP7 is a Dub of RNF6, we next analyzed the polyubiquitination levels of RNF6 after P5091 treatment by using the IP/IB assay. The results showed that P5091 increased RNF6 polyubiquitination in a concentration-dependent manner (Fig. 5*C*). Specifically, P5091 markedly increased RNF6 at the K48-linked polyubiquitination form (Fig. 5*D*), in association with its degradation (Fig. 5*A*). And this finding was further confirmed by the CHX chase assay in which when protein synthesis *de novo* was inhibited by CHX, P5091 strikingly increased the turnover rate of RNF6 and the half-life of RNF6 was significantly reduced (Fig. 5*E*). Notably, P5091 displayed no activity toward the stability of ΔRNF6 or RNF6^C/S^ but the RNF6 (Fig. 5*F*). Therefore, all these results collectively concluded that USP7 was a Dub of RNF6 auto-ubiquitination and a stabilizer of RNF6 protein.

### Chemical induction of RNF6 auto-ubiquitination to induce MM and leukemia cell apoptosis

**Figure 6:**
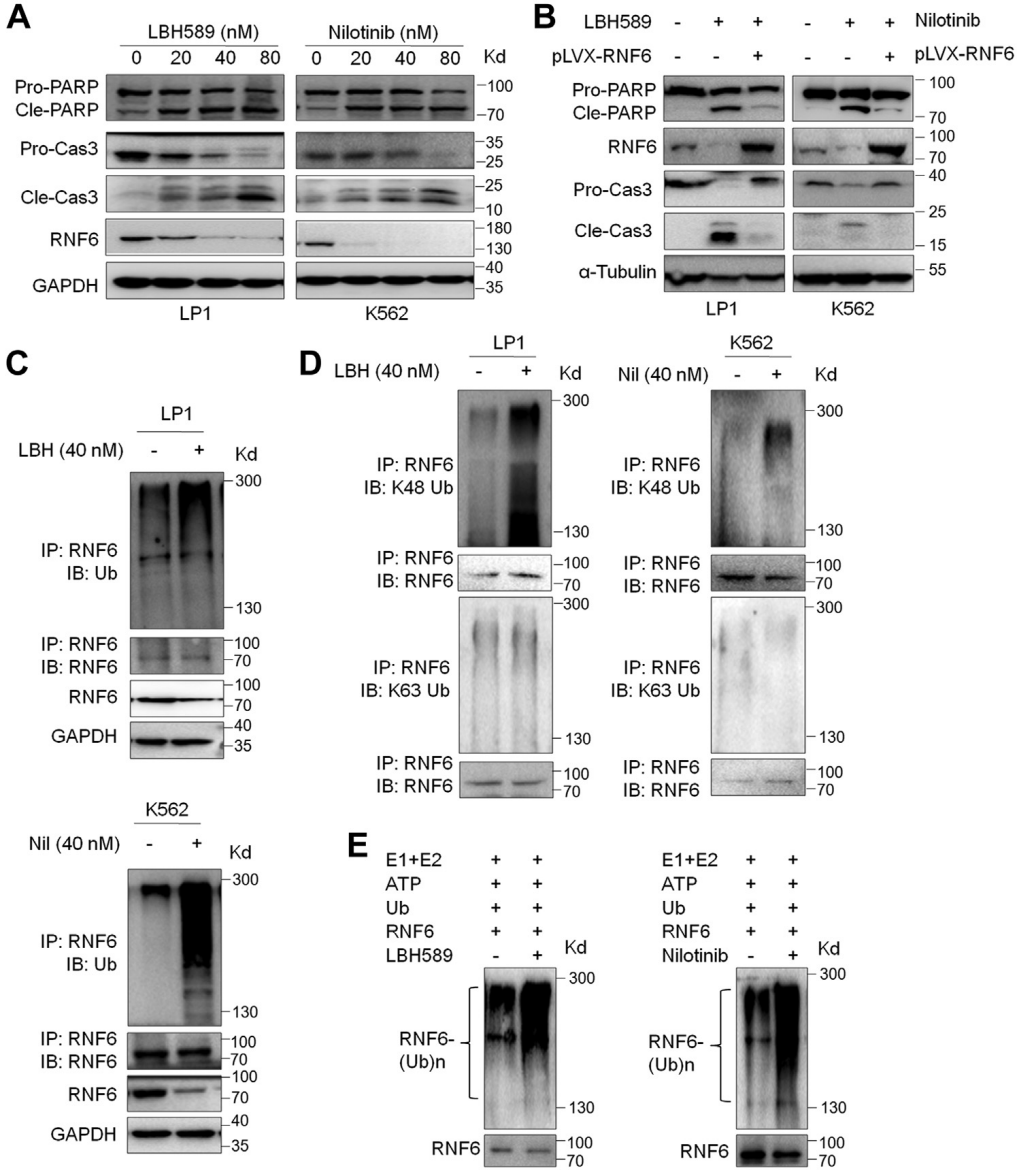
**Anti-cancer drugs induce RNF6 auto-ubiquitination and degradation.***A*, LP1 and K562 cells were treated overnight with LBH589 and Nilotinib, respectively. The cell lysates were then subjected to IB assays with specific antibodies as indicated. *B*, LP1 and K562 cells were infected with RNF6-carrying lentivirus for 72 h, followed by LBH589 and Nilotinib treatment, respectively, for 24 h. Cell lysates were then prepared for an IB assay. *C* and *D*, LP1 and K562 cells were treated with LBH589 or Nilotinib for 24 h, respectively, followed by an IP/IB assay to examine the ubiquitination type. *E*, purified RNF6 protein was treated with Nilotinib or LBH589 for 1 h, followed by incubation with reaction buffer containing HA-Ub, E1, E2, and ATP for 2 h. After reaction was terminated, the mixture was subjected to IP/IB assays. IB, immunoblot; IP, immunoprecipitation.

Given that the downregulation of RNF6 protein could trigger MM and leukemia cell apoptosis (1, 2, 3) and auto-ubiquitination could lead to RNF6 degradation, we wondered whether induction of RNF6 auto-ubiquitination and degradation could lead to MM and leukemia cell death. To this end, a panel of anti-MM and anti-leukemia drugs including Panobinostat (LBH589) (10), an FDA-approved anti-MM drug, and Nilotinib, an FDA-approved anti-leukemia drug (11), were applied for the study. As shown in Figure 6*A*, LBH589 and Nilotinib induced apoptosis of MM cell line LP1 and leukemia cell line K562, respectively, as evidenced by the cleavage of PARP and Caspase-3, hallmarks of cell apoptosis, which was consistent with previous studies (12, 13). To find out whether RNF6 was involved in cell apoptosis induced by these two drugs, LP1 and K562 cells were infected with lentiviral RNF6, followed by drug treatment and IB assays. The results showed that both LBH589 and Nilotinib induced cell apoptosis as evidenced by PARP cleavage and Caspase-3 activation (Fig. 6*B*). However, when RNF6 was overexpressed, both PARP cleavage and Caspase-3 activation were markedly reduced (Fig. 6*B*), suggesting RNF6 partly abolished apoptosis induced by these two drugs and that RNF6 was a potential target of the drugs. Next, we wondered whether this kind of degradation of RNF6 was associated with ubiquitination. To find this out, the proteins from drug-treated cells were subjected to IP with an anti-RNF6 antibody followed by ubiquitination evaluation by IB assays with a ubiquitin-specific antibody. It clearly demonstrated that both drugs markedly increased RNF6 polyubiquitination (Fig. 6*C*). Moreover, we found that both LBH589 and Nilotinib specifically induced the K48- but not the K63-linked form (Fig. 6*D*). Furthermore, we found that these drugs directly mediated RNF6 polyubiquitination in the *in vitro* assay in the absence of any extra ubiquitination ligases (Fig. 6*E*). Therefore, these results together firmly demonstrated that RNF6 auto-ubiquitination could be activated by clinical drugs such as LBH589 and Nilotinib. It also suggests that chemical induction of RNF6 auto-ubiquitination could be a novel therapeutic strategy against hematological malignancies such as myeloma and leukemia.

## Discussion

Auto-ubiquitination is a characteristic of the RING E3 ligases (4), and auto-ubiquitination may represent a significant manner by which E3 ligases regulate their own stability within the cell. Induction of auto-ubiquitination and subsequent degradation could be a potential strategy for cancer treatment by targeting a specific oncogenic RING family E3 ligase (14). Therefore, understanding the molecular modulation is critical to develop novel RING E3 ligases as targets for cancer treatment. In the present study, we provided comprehensive evidence that the RING family ubiquitin ligase RNF6 undergoes auto-ubiquitination, and induction of RNF6 auto-ubiquitination could be a promising strategy for the treatment of some hematological malignancies such as MM and leukemia.

RNF6 as a ubiquitin ligase has been reported in various cancers that modifies substrate proteins, therefore promoting their oncogenic activity. For example, RNF6 mediates K27-linked polyubiquitination of the androgen receptor (AR), thereby recruiting the chaperone proteins to increase the oncogenic transcriptional activity of AR in prostate cancer (15). In myeloma cells, RNF6 binds to and triggers glucocorticoid receptor for K63-linked polyubiquitination, therefore promoting its transcriptional activity to promote the transcription of prosurvival genes including Bcl2L1 and Mcl-1 (1). RNF6 also acts on some tumor suppressors such as SHP-1 (16) and TLE3 (17) by promoting their polyubiquitination and degradation in colorectal cancer. In the present study, we found that RNF6 as a RING family E3 ligase can direct its own polyubiquitination, and this action depends on its RING domain. Both the *in vivo* and the *in vitro* ubiquitination assays demonstrate that when the RING domain is deleted, RNF6 loses its auto-ubiquitination ability. It is well known that the cysteine residue cooperates with histidine residues in the RING domain to act as ligands of Zn^2+^ to stabilize the scaffold of the RING finger E3 ligase (18). We indeed found that the C/S mutant becomes significantly stable; however, we also found that in the extended treatment (24 h), MG132 can increase RNF6^C/S^ stability, which is consistent with the CHX assay, suggesting that, different from the ΔRING mutant, the mutant RNF6^C/S^ probably could be degraded *via* the proteasomes. But this finding does not occlude the conclusion that loss of either RING or C2H2 structure inactivates RNF6 in its ubiquitin ligase activity because in the absence of any extra E3 ligases in the *in vitro* ubiquitination assay, only the WT but not the ΔRING or C/S mutant could be polyubiquitinated, suggesting that RNF6 undergoes auto-polyubiquitination and this auto-ubiquitination depends on its RING domain. This finding is consistent with previous reports on protein auto-ubiquitination, such as XIAP (5), TRIM26 (7), and others.

In the present study, we also found that RNF6 auto-ubiquitination could be prevented by the deubiquitinase USP7 which was found from an affinity purification–coupled tandem mass spectrometry targeting RNF6-interacting proteins. Although both USP7 and USP9x were found in the interacting complexes of RNF6, only USP7 but not USP9x prevents RNF6 auto-ubiquitination. Interestingly, it is reported that both USP7 and RNF6 bind to AR proteins; one might doubt whether this interaction between USP7 and RNF6 was indirect and AR is probably a linker between them. However, as demonstrated in our previous study, MM cells do not express AR (1), but USP7 and RNF6 bind to each other as evidenced by the reciprocal IP/IB assays (Fig. 2, *D* and *E*). Consistent with RNF6 auto-ubiquitination, USP7 dose-dependently reduces K48-linked but not other forms of polyubiquitination on RNF6, which is consistent with the modulation of USP7 on RNF6 protein stability but not its mRNA level. Interestingly, in addition to our current study, USP7 has been found to interfere with auto-ubiquitination of other proteins, including ICP0 (4), Chfr (19), and UHRF1 (20). Consistent with the action of USP7 on RNF6 auto-ubiquitination and stability, inhibition of USP7 by a chemical or genetic manner could trigger RNF6 degradation and associated cell death (Fig. 5).

Moreover, RNF6 could undergo polyubiquitination at a baseline level, suggesting the stability of RNF6 itself is strictly tuned, probably by itself, the Dub USP7, and other unknown factors. We also found that RNF6 is highly expressed in hematological malignancies including chronic leukemia (2), acute leukemia (6), and myeloma (1), suggesting that RNF6 auto-ubiquitination and proteasomal degradation are dysregulated or suppressed. This hypothesis is confirmed by the inhibition of USP7. Both genetic and chemical inhibition of USP7 can restore RNF6 auto-ubiquitination and lead to RNF6 degradation. Interestingly, we found that anti-MM LBH589 and anti-leukemia Nilotinib can also induce RNF6 auto-polyubiquitination in the *in vitro* ubiquitination system that lacks any other enzymes but recombinant human His6-Ubiquitin E1 enzyme (UBE1) and recombinant human UbcH5a/UBE2D1 protein as described by the manufacturer (Boston Biochem Inc). Although the detailed mechanism is not yet known, the auto-ubiquitination induced by both drugs is confirmed in both cells and in the cell-free system. Notably, both drugs induce RNF6 degradation and the reexpression of RNF6 can partly ablate the action of both drugs in terms of PARP cleavage, the hallmark of cell apoptosis. These findings thus suggest that RNF6 might also be a direct target of LBH589 and Nilotinib. It is well known that LBH589 is a HDAC inhibitor that increases the acetylation level of some proteins such as histone H3 and H4, therefore promoting the transcription of tumor suppressor genes, such as p21 and p27. Nilotinib is an inhibitor of multiple kinases including BCR-ABL; therefore, it is used for the treatment of refractory and resistant patients with CML. Our current findings add a secondary pharmacological activity to these drugs in addition to their primary one. The significance of the present study is that triggering RNF6 auto-ubiquitination by certain chemical compounds could be a potential novel method to induce MM and leukemia cell death.

In summary, the present study demonstrates that RNF6 undergoes auto-ubiquitination, and USP7 is a Dub of RNF6 auto-ubiquitination. Moreover, we found that induction of RNF6 auto-ubiquitination and proteasomal degradation leads to myeloma and leukemia cell death. This study thus establishes a rationale that targeting auto-ubiquitination of oncogenic RING-domain containing ubiquitin ligases by chemical drugs could be a novel strategy for the treatment of hematological malignancies, such as myeloma and leukemia.

## Experimental procedures

### Cells and cell culture

MM cell lines (RPMI-8226, LP1, MM1.S, OPM2, KMS11, and OCI-My5) and leukemia cell lines (K562, OCI-AML2, Jurkat, KBM5, and NB4) were purchased from the American Type Culture Collection (ATCC) or generously provided by Dr Aaron D. Schimmer (Princess Margaret Cancer Center). HEK293 and HEK293T cells were maintained in Dulbecco’s modified Eagle’s medium. All cells were cultured in 10% fetal bovine serum (ExCell Biotech Co, Ltd) and appropriate antibiotics. Bone marrow cells were collected with written consent from myeloma patients who volunteered to donate bone marrow for research purpose from Department of Hematology, Affiliated Hospital of Guangdong Medical University. The studies in this work abide by the Declaration of Helsinki principles and were approved by the Review Board and Ethic Committee of the Affiliated Hospital of Guangdong Medical University.

### Plasmids

A V5-USP9X plasmid was cloned as described previously (21). The plasmids for USP7 and truncates were generated as described previously (22). The lentiviral USP7 and sgUSP7 plasmids were prepared as reported previously (23). The RNF6 coding sequences were obtained from the NCBI website (http://www.ncbi.nlm.nih.gov) and subcloned into a pcDNA3.1 vector carrying a Flag or a Myc tag. The RING domain deleting RNF6 (ΔRING) was amplified by PCR with primers as follows: Forward 5′-CCCGGAATTCATGAATCAGTCTAGATCGAG-3′ and Reverse 5′-AAATATGCGGCCGCGATTTTACCTAGTTCAC-3′. The RNF6-C632/635S mutant was created by site-directed mutagenesis using WT RNF6 as the template and the specific primers as follows: Forward 5′-CtctagtgtttctATTAGTGACTATGTAACTGGAAACAAGCT-3′ and Reverse 5′-CTAATagaaacactagaGATTTTACCTAGTTCACTATCAATACTGTTATG-3′. The site-directed mutagenesis was achieved by using the Mut Express II Fast Mutagenesis Kit V2 (Vazyme Biotech Co, Ltd), according to the instructions of the manufacturer (24).

### Antibodies and chemicals

Antibodies against USP7 (Cat. CST#4833), K48Ub (Cat. CST#4289), and K63Ub (Cat. CST#12930) were purchased from Cell Signaling Technologies, Inc. The antibodies against RNF6 (Cat. #20437-1-AP), USP9x (Cat. #55054-1-AP), PARP (Cat. #13371-1-AP), Caspase 3 (Cat. #19677-1-AP), GAPDH (Cat. #60004-1-Ig), α-tubulin (Cat. #11224-1-AP), and V5 (Cat. #14440-1-AP) were obtained from Proteintech. The monoclonal antibodies including anti-Flag (Cat. #M185-3L), anti-HA (Cat. #M180-3), and anti-Myc (Cat. #M192-3) were obtained from Medical and Biological Laboratories Co Ltd. The anti-Ub antibody (Cat. #SL-8017) was purchased from Santa Cruz Biotechnology, Inc. HRP-labeled goat anti-mouse (Cat. #A0216) and goat anti-rabbit IgG (H + L) antibodies (Cat. #A0208) were purchased from Beyotime Institute of Biotechnology. MG132 (Cat. #S2619), Bortezomib (Cat. #S1013), and P5091 (Cat. #S7132) were purchased from Santa Cruz Biotechnology and Selleck Chemicals Inc, respectively. CHX (Cat. #C7698) and chloroquine (CHQ, Cat. #C6628) were purchased from Sigma-Aldrich. LBH589 (LBH, Cat. #M1748) was purchased from AbMole Bioscience Inc. Nilotinib (Cat. #IN0560) was purchased from Solarbio Life Science. Recombinant ubiquitin (Cat. #U100-H), UBE1 (Cat. #E-304), UBE2D1(Cat. #E2-616), and ATP (Cat. #B-20) were all purchased from Boston Biochem Inc.

### Reverse-transcription polymerase chain reaction

Total RNA was extracted using Trizol (TransGen Biotech Co, Ltd). RNA (2.5 μg) was reverse-transcribed using the EasyScipt First-strand cDNA Synthesis SuperMix (TransGen Biotech), according to the manufacturer’s instructions. PCR amplification was performed using the following primers: for USP7, Forward 5′-TTTTGTGCGAAATCTGCC-3′ and Reverse 5′-AATCCCACGCAACTCCAT-3′; for RNF6, Forward 5′-CATCAGTGGCTCTTCGGTCA-3′ and Reverse5′-ATGCTCATAGTGCCTGGTGG-3′; for GAPDH, Forward 5′-AGTCCACTGGCGTCTTCA-3′ and Reverse 5′-CTCCGACGCCTGCTTCACCA-3′. Reaction cycling conditions were 3 min at 95 °C, followed by 30 cycles at 95 °C for 30 s, 60 °C for 30 s, and 72 °C for 40 s, and 1 cycle at 72 °C for 10 min. Products were analyzed on 2% agarose gels (TransGen Biotech). The PCR products were visualized by Goldview staining (TransGen Biotech) following electrophoresis on 2% agarose gels.

### RNF6 and USP7 truncates

The USP7 truncates were constructed previously (22). To generate RNF6 truncates, we used the following primers: for RNF6 (1–482), Forward 5′-cttggtaccgagctcggatccATGAATCAGTCTAGATCGAGATCAGATG-3′, Reverse 5′-ttcgggccctctagactcgagTGACCGAAGAGCCACTGATGA-3′; for RNF6 (1–632), Forward 5′-cttggtaccgagctcggatccATGAATCAGTCTAGATCGAGATCAGATG-3′, Reverse 5′-ttcgggccctctagactcgagACAGATTTTACCTAGTTCACTATCAATACTG-3′; for RNF6 (483–685), Forward 5′-cttggtaccgagctcggatccATTTTAAGGCAGATCATGACTGGG-3′, Reverse 5′-ttcgggccctctagactcgagCCCATTGTTTGCTATGTTAGACCC-3′; for RNF6 (87–482), Forward 5′-cttggtaccgagctcggatccGACTTGAGAGATGGAACGAATTACAG-3′, Reverse 5′-ttcgggccctctagactcgagTGACCGAAGAGCCACTGATGA-3′. The reaction was performed using full-length RNF6 as the template.

### Gene transfection and viral infection

HEK293T cells at 50% confluence were subjected to gene transfection using PEI as the carrier as described previously (25). The viral particles were prepared with a standard method according to the manufacturer’s instructions and included control and package plasmids (Shanghai GeneChem Co Ltd). The individual plasmids were cotransfected into HEK293T cells with the calcium precipitate method. Viruses were obtained 72 h later from transfected cells. All viruses were filtered *via* a 0.45 μm membrane and concentrated before being applied to infect the appropriate cells.

### Immunoblot

Prepared proteins were fractionated in sodium dodecyl sulfate–polyacrylamide gel electrophoresis (SDS-PAGE), the proteins were then transferred to PVDF membranes (Millipore). After being pre-blotted in 5% milk, the blots were then incubated with primary antibodies of interest overnight at 4 °C. The blots were then processed as described previously (2).

### Immunoprecipitation

Cell lysates from cells of interest were prepared in a protein cell lysis buffer (25). After clarification and concentration determination, an equal number of total proteins were incubated with specific antibodies overnight at 4 °C followed by incubation with Protein A + G beads (Beyotime Biotechnology) for 2 h at room temperature. After washing, the protein-containing beads were collected and boiled in 2× SDS loading dye before being subjected to IB assays.

### Affinity purification–coupled tandem mass spectrometry assay

HEK293T cells were transfected with a Flag–RNF6ΔRING plasmid or empty vector for 36 h before being treated with MG132 (10 μM) for another 4 h. The cells were then collected for protein extraction using a lysis buffer as described previously (25). After lysis, the clarified cell lysates (10 mg of each sample) were subjected to co-IP using anti-Flag M2 Affinity Gel (Sigma-Aldrich) overnight at 4 °C. After a gentle washing, proteins were separated by SDS-PAGE followed by silver staining (Beyotime InstituteBiotechnology). The protein bands from the gel were excised and processed for HPLC and tandem mass spectrometry analysis as described previously (25).

### Mass spectrometry data analysis

Tandem mass spectrometry raw files were analyzed by using Proteome Discoverer (version 1.4). The Andromeda probabilistic search engine was used to search peak lists against the UniProt database (released in July 2017, containing 182,230 entries). Cysteine carbamidomethylation was set as a fixed modification and methionine oxidation was set as a variable modification. The digestion enzyme was set as trypsin, and a maximum of two missed-cleavage sites were allowed. A minimum of seven amino acids per identified peptide were required. The mass tolerance for precursor ions was set to 20 ppm for the first search and 4.5 ppm for the main search and that for fragment ions was set to 0.5 Da. The false discovery rate was determined by searching a reverse database and was set as 1% for proteins and peptides.

### In-tube ubiquitination assay

Flag-RNF6-WT, Flag-RNF6-ΔRING, and Flag-RNF6-C/S proteins were purified with specific antibodies against Flag as described previously (26). These proteins were then added to the reaction mixture containing ATP, HA-Ub, E1, and E2 (Boston Biochem Inc) for ubiquitination according to manufacturer’s instruction (25). For the in-tube ubiquitination assay induced by chemical drugs, purified Flag-RNF6 protein was first preincubated with 40 nM of LBH589 or Nilotinib for 1 h, followed by incubation with the above reaction system for 2 h. After the reaction was terminated, the mixture was subjected to IB assays.

### CHX chase assay

After transfection with plasmids of interest for 24 h, HEK293T cells were treated with 100 μg/ml of cycloheximide (CHX. Cat.# c7698, Sigma-Aldrich) for periods of interest. Cell lysates were then prepared for SDS-PAGE and IB analyses with specific antibodies.

### Densitometric analyses

Densitometric analyses of Western blots in the terms of protein stability were performed as described previously (23), using ImageJ software developed by the National Institutes of Health.

### Statistical analysis

Student’s *t* test was used for comparisons between two groups in the study. All statistical tests were two-sided, and a *p* value <0.05 indicated statistical significance.

## Data availability

The mass spectrometry proteomics raw data have been deposited to the ProteomeXchange Consortium (http://proteomecentral.proteomexchange.org) *via* the iProX partner repository (27) with the dataset identifier PXD032990.

## Conflict of interest

All the authors declare that they have no conflict of interest related to this study.

## References

[1] Ren Y., Xu X., Mao C.Y., Han K.K., Xu Y.J., Cao B.Y. (2020). Rnf6 promotes myeloma cell proliferation and survival by inducing glucocorticoid receptor polyubiquitination. Acta Pharmacol. Sin..

[2] Xu X., Han K., Tang X., Zeng Y., Lin X., Zhao Y. (2016). The ring finger protein rnf6 induces leukemia cell proliferation as a direct target of pre-B-cell leukemia homeobox 1. J. Biol. Chem..

[3] Lu Q., He Y., Wang Y., Gao L., Zheng Y., Zhang Z. (2018). Saponins from Paris forrestii (Takht.) H. Li display potent activity against acute myeloid leukemia by suppressing the RNF6/AKT/mTOR signaling pathway. Front. Pharmacol..

[4] Canning M., Boutell C., Parkinson J., Everett R.D. (2004). A RING finger ubiquitin ligase is protected from autocatalyzed ubiquitination and degradation by binding to ubiquitin-specific protease USP7. J. Biol. Chem..

[5] Yang Y., Fang S., Jensen J.P., Weissman A.M., Ashwell J.D. (2000). Ubiquitin protein ligase activity of IAPs and their degradation in proteasomes in response to apoptotic stimuli. Science.

[6] Park Y., Pang K., Park J., Hong E., Lee J., Ooshima A. (2020). Destablilization of TRAF6 by DRAK1 suppresses tumor growth and metastasis in cervical cancer cells. Cancer Res..

[7] Ran Y., Zhang J., Liu L.L., Pan Z.Y., Nie Y., Zhang H.Y. (2016). Autoubiquitination of TRIM26 links TBK1 to NEMO in RLR-mediated innate antiviral immune response. J. Mol. Cell Biol..

[8] Ma J., Martin J.D., Xue Y., Lor L.A., Kennedy-Wilson K.M., Sinnamon R.H. (2010). C-terminal region of USP7/HAUSP is critical for deubiquitination activity and contains a second mdm2/p53 binding site. Arch. Biochem. Biophys..

[9] Chauhan D., Tian Z., Nicholson B., Kumar K.G., Zhou B., Carrasco R. (2012). A small molecule inhibitor of ubiquitin-specific protease-7 induces apoptosis in multiple myeloma cells and overcomes bortezomib resistance. Cancer Cell.

[10] Laubach J.P., Tuchman S.A., Rosenblatt J.M., Mitsiades C.S., Colson K., Masone K. (2021). Phase 1 open-label study of panobinostat, lenalidomide, bortezomib + dexamethasone in relapsed and relapsed/refractory multiple myeloma. Blood Cancer J..

[11] Jain P., Kantarjian H., Alattar M.L., Jabbour E., Sasaki K., Nogueras Gonzalez G. (2015). Long-term molecular and cytogenetic response and survival outcomes with imatinib 400 mg, imatinib 800 mg, dasatinib, and nilotinib in patients with chronic-phase chronic myeloid leukaemia: retrospective analysis of patient data from five clinical trials. Lancet Haematol..

[12] Maiso P., Carvajal-Vergara X., Ocio E.M., Lopez-Perez R., Mateo G., Gutierrez N. (2006). The histone deacetylase inhibitor LBH589 is a potent antimyeloma agent that overcomes drug resistance. Cancer Res..

[13] Sacha T., Saglio G. (2019). Nilotinib in the treatment of chronic myeloid leukemia. Future Oncol..

[14] Yang Y.L., Li X.M. (2000). The iap family: endogenous caspase inhibitors with multiple biological activities. Cell Res..

[15] Xu K., Shimelis H., Linn D.E., Jiang R., Yang X., Sun F. (2009). Regulation of androgen receptor transcriptional activity and specificity by RNF6-induced ubiquitination. Cancer Cell.

[16] Liang Q., Ma D., Zhu X., Wang Z., Sun T.T., Shen C. (2018). RING-finger protein 6 amplification activates JAK/STAT3 pathway by modifying SHP-1 ubiquitylation and associates with poor outcome in colorectal cancer. Clin. Cancer Res..

[17] Liu L., Zhang Y., Wong C.C., Zhang J., Dong Y., Li X. (2018). RNF6 promotes colorectal cancer by activating the Wnt/beta-catenin pathway via ubiquitination of TLE3. Cancer Res..

[18] Cassandri M., Smirnov A., Novelli F., Pitolli C., Agostini M., Malewicz M. (2017). Zinc-finger proteins in health and disease. Cell Death Discov..

[19] Oh Y.M., Yoo S.J., Seol J.H. (2007). Deubiquitination of Chfr, a checkpoint protein, by USP7/HAUSP regulates its stability and activity. Biochem. Biophys. Res. Commun..

[20] Ahmad T., Ashraf W., Ibrahim A., Zaayter L., Muller C.D., Hamiche A. (2021). TIP60 governs the autoubiquitination of UHRF1 through USP7 dissociation from the UHRF1/USP7 complex. Int. J. Oncol..

[21] Zhang Y., Duan C., Yang J., Chen S., Liu Q., Zhou L. (2018). Deubiquitinating enzyme USP9X regulates cellular clock function by modulating the ubiquitination and degradation of a core circadian protein BMAL1. Biochem. J..

[22] He Y., Wang S., Tong J., Jiang S., Yang Y., Zhang Z. (2020). The deubiquitinase USP7 stabilizes Maf proteins to promote myeloma cell survival. J. Biol. Chem..

[23] Jiang S., Wang X., He Y., Huang H., Cao B., Zhang Z. (2021). Suppression of USP7 induces BCR-ABL degradation and chronic myelogenous leukemia cell apoptosis. Cell Death Dis..

[24] Xu Y., Xu M., Tong J., Tang X., Chen J., Chen X. (2021). Targeting the Otub1/c-Maf axis for the treatment of multiple myeloma. Blood.

[25] Zhang Z., Tong J., Tang X., Juan J., Cao B., Hurren R. (2016). The ubiquitin ligase HERC4 mediates c-Maf ubiquitination and delays the growth of multiple myeloma xenografts in nude mice. Blood.

[26] Huang X., Zhang Q., Lou Y., Wang J., Zhao X., Wang L. (2019). USP22 deubiquitinates CD274 to suppress anticancer immunity. Cancer Immunol. Res..

[27] Ma J., Chen T., Wu S., Yang C., Bai M., Shu K. (2019). iProX: an integrated proteome resource. Nucleic Acids Res..

